# Engagement, Satisfaction, and Mental Health Outcomes Across Different Residential Subgroup Users of a Digital Mental Health Relational Agent: Exploratory Single-Arm Study

**DOI:** 10.2196/46473

**Published:** 2023-09-27

**Authors:** Valerie L Forman-Hoffman, Maddison C Pirner, Megan Flom, Andrew Kirvin-Quamme, Emily Durden, Jennifer A Kissinger, Athena Robinson

**Affiliations:** 1 Woebot Health San Francisco, CA United States

**Keywords:** adoption, anxiety, chatbot, cognitive behavioral therapy, conversational agent, CBT, depression, digital health, medically underserved area, mental health, mhealth, mobile app, mobile health, mobile phone, mood, psychotherapy, relational agent, rural, satisfaction, smartphone app, smartphone, underserved, usage, vulnerable

## Abstract

**Background:**

Mental illness is a pervasive worldwide public health issue. Residentially vulnerable populations, such as those living in rural medically underserved areas (MUAs) or mental health provider shortage areas (MHPSAs), face unique access barriers to mental health care. Despite the growth of digital mental health interventions using relational agent technology, little is known about their use patterns, efficacy, and favorability among residentially vulnerable populations.

**Objective:**

This study aimed to explore differences in app use, therapeutic alliance, mental health outcomes, and satisfaction across residential subgroups (metropolitan, nonmetropolitan, or rural), MUAs (yes or no), and MHPSAs (yes or no) among users of a smartphone-based, digital mental health intervention, Woebot LIFE (WB-LIFE). WB-LIFE was designed to help users better understand and manage their moods and features a relational agent, Woebot, that converses through text-based messages*.*

**Methods:**

We used an exploratory study that examined data from 255 adults enrolled in an 8-week, single-arm trial of WB-LIFE. Analyses compared levels of app use and therapeutic alliance total scores as well as subscales (goal, task, and bond), mental health outcomes (depressive and anxiety symptoms, stress, resilience, and burnout), and program satisfaction across residential subgroups.

**Results:**

Few study participants resided in nonmetropolitan (25/255, 10%) or rural (3/255, 1%) areas, precluding estimates across this variable. Despite a largely metropolitan sample, nearly 39% (99/255) resided in an MUA and 55% (141/255) in an MHPSA. There were no significant differences in app use or satisfaction by MUA or MHPSA status. There also were no differences in depressive symptoms, anxiety, stress, resilience, or burnout, with the exception of MUA participants having higher baseline depressive symptoms among those starting in the moderate range or higher (Patient Health Questionnaire-8 item scale≥10) than non-MUA participants (mean 16.50 vs 14.41, respectively; *P*=.01). Although working alliance scores did not differ by MHPSA status, those who resided in an MUA had higher goal (2-tailed *t*_203.47_=2.21; *P*=.03), and bond (*t*_203.47_=1.94; *P*=.05) scores at day 3 (*t*_192.98_=2.15; *P*=.03), and higher goal scores at week 8 (*t*_186.19_=2.28; *P*=.02) as compared with those not living in an MUA.

**Conclusions:**

Despite the study not recruiting many participants from rural or nonmetropolitan populations, sizable proportions resided in an MUA or an MHPSA. Analyses revealed few differences in app use, therapeutic alliance, mental health outcomes (including baseline levels), or satisfaction across MUA or MHPSA status over the 8-week study. Findings suggest that vulnerable residential populations may benefit from using digital agent–guided cognitive behavioral therapy.

**Trial Registration:**

ClinicalTrials.gov NCT05672745; https://clinicaltrials.gov/study/NCT05672745

## Introduction

Mental illness is a pervasive worldwide public health issue that accounts for 7% of the overall global burden of disease [1], with depression being the leading cause of disability [2]. Incidence rates continue to increase, with both depression and anxiety increasing by nearly 25% globally since the COVID-19 pandemic [3]. The capacity issues of face-to-face services cannot meet the demand for care [4].

Residentially vulnerable populations, such as residents of rural areas, medically underserved areas (MUAs), and critical mental health provider shortage areas (MHPSAs), are particularly limited in their access to effective mental health treatment [5]. Approximately 60% of individuals with a psychiatric disorder residing in MUAs and MHPSAs are treated solely by primary care physicians [6], who are often the only treatment source for rural populations [7,8]. Mental health treatment in these areas is fragmented, inconsistent, and largely inadequate, with only 13% of care provided by primary care physicians considered even “minimally adequate” [6-8]. Digital mental health interventions (DMHIs) have tremendous potential to mitigate mental health disparities and revolutionize access to mental health care.

Research investigating DMHIs has expanded rapidly in recent years [9]. Evidenced to effectively treat a variety of mental health conditions [10-15], DMHIs are being increasingly integrated into European and Australian health care systems. They present a scalable solution for optimizing the efficiency and accessibility of effective mental health services in the United States, where approximately 150 million people live in areas experiencing shortages of mental health providers [16].

Although evidence shows promise for equal efficacy and acceptability of DMHIs among users in both rural and urban areas [17,18], studies of mental health relational agents that have demonstrated preliminary efficacy and effectiveness [19,20] have not investigated residential inequities. Thus, large-scale studies examining the efficacy, acceptability, and feasibility of such programs across underserved residential populations are necessary.

Woebot LIFE (WB-LIFE) is a smartphone-based DMHI app that enlists the relational agent Woebot to guide users in managing their moods through text-based messages. Previous research has found Woebot-based DMHIs to be feasible, acceptable, and efficacious among various populations [19,21]. This study adopted an exploratory approach to investigate differences in app use, therapeutic alliances, mental health outcomes, and satisfaction across different residential subgroups of WB-LIFE users.

## Methods

### Study Description

This study explored data from an 8-week single-armed trial of WB-LIFE. The purpose of this study was to explore differences in various key outcomes (app use, therapeutic alliance, mental health outcomes, and satisfaction) across different residential subgroups of users. US adults were recruited through social media advertisements over a 10-day period in May 2022. Following a screening and baseline survey, enrolled participants had access to WB-LIFE and received emailed follow-up assessments at day 3, week 4, and week 8 postbaseline.

### Survey and App Use Measures

A dichotomous app use metric indicated if a participant opened the app at least half of the study weeks (4/8). The Working Alliance Inventory-Short Revised assessed therapeutic alliance [22] on day 3 and week 8 through a total score and 3 subscales—goal, task, and bond. The Client Satisfaction Questionnaire [23] assessed satisfaction at week 8. Depressive symptoms, anxiety symptoms, stress, resilience, and burnout measurements included the Patient Health Questionnaire-8 item scale (PHQ-8) [24], Generalized Anxiety Disorder-7 item scale (GAD-7) [25], Perceived Stress Scale [26], Brief Resilience Scale [27], and a nonproprietary single-item measure of burnout [28] dichotomized in the literature into having symptoms of burnout (response category 3, 4, or 5) versus having no symptoms of burnout (response category 1 or 2) [29], respectively.

### Rural, Urban, Health Professional Shortage Area

Participants provided a residential address with consent. Addresses were matched to the corresponding health professional shortage area status according to designations by the Health Resources and Services Administration, US Department of Health and Human Services [30] to obtain MHPSA and MUA classifications. The Health Resources and Services Administration database [30] provided a county-specific Federal Information Processing System code used to verify the participant’s residential location and status on a rural-urban continuum code [31]. The rural-urban continuum codes use US Census Bureau County designations to classify residential locations onto a 9-code scale of metropolitan (3 categories), nonmetropolitan (4 categories), and rural (2 categories) based on county population size, degree of urbanization, and proximity to a metropolitan area [31-33].

### Data Analysis

The Working Alliance Inventory-Short Revised analysis used day 3 and week 8 scores. All other analyses focused on each mental health construct measured at baseline, week 8, and the change score at week 8.

The 2-tailed *t* tests or ANOVAs compared mean differences across residential groups, with 2 exceptions. Analyses of categorical measures of burnout and app use used chi-square tests. For PHQ-8 and GAD-7 outcomes only (ie, baseline, week 8, and change at week 8), residential group means were compared in both the overall sample and the sample with at least moderate levels of baseline score PHQ-8 ≥10 or GAD-7 ≥10, which have been validated as indicative of clinical levels of major depressive disorder and generalized anxiety disorder, respectively [24,25]. Analyses of any variable deemed unstable, defined as having 1 or more cell sizes of 15 or less, are not reported herein. Because of the exploratory nature of this paper, we did not adjust for multiple comparisons and have focused our conclusions on overall patterns.

### Ethics Approval

The study was approved by the Western Institutional Review Board-Copernicus Institutional Review Board group on January 20, 2022 (Protocol #20216751); all participants provided informed consent. See [34] for a detailed description of the trial procedure, including the inclusion and exclusion criteria, the safety assessment protocol, and the intervention.

## Results

Although 256 participants enrolled in the study, analyses focused on 255 because 1 participant did not provide residence data to allow for residential classifications of interest. Few study participants (28/255, 11%) resided in a nonmetropolitan (population 2500-49,999; n=25) or rural (population <2500; n=3) area, which precluded reporting estimates across these strata. Despite a largely metropolitan sample (population >50,000), nearly 40% (99/255) lived in an MUA and 55% (141/255) in an MHPSA.

As seen in Table S1 in Multimedia Appendix 1, *t* tests indicated no mean differences in app use, satisfaction, or 8-week depressive symptoms, anxiety symptoms, stress, resilience, or burnout variables by MUA or MHPSA. Baseline levels of symptoms also did not differ across groups, with 1 exception. For those with clinically elevated depression at baseline (PHQ-8≥10), those who did not live in an MUA had lower baseline depressive symptom scores than those who did (*P*=.01; Cohen *d*=−0.52), although differences did not persist at week 8. Lastly, Working Alliance Inventory scores did not differ by MHSPS status at day 3 or week 8. Those who did not live in an MUA, however, had lower Working Alliance Inventory goal (*P*=.03), bond (*P*=.05), and total scores at day 3 (*P*=.03) and lower goal at week 8 (*P*=.02) than those living in an MUA, although effect sizes were relatively small (Cohen *d*=−0.29, −0.25, −0.28, and −0.31, respectively).

## Discussion

Despite the study not recruiting many participants from rural or nonmetropolitan populations, sizable proportions resided in an MUA (99/255, 39%) or an MHPSA (141/255, 55%). The small sample size of the rural population precluded the reporting of study findings for this group. Analyses across MHPSA status revealed no significant differences for nearly all outcomes; however, there were a few differences in therapeutic alliance and baseline depressive symptoms across MUA status. First, among those with clinical levels of symptomatology at baseline (PHQ-8 ≥10), those living in an MUA had higher levels of baseline depressive symptoms. Second, those living in an MUA had several higher therapeutic alliance scores than those who did not. Thus, despite differential depressive symptoms at baseline consistent with previous findings of greater symptomatology and treatment need in residentially inequitable subgroups [35,36], the levels of therapeutic alliance formed by those in MUAs appeared similar to or even greater than those not living in MUAs.

Although this is the first published report focused on whether app use, therapeutic alliance, mental health outcomes, and satisfaction differ across residentially inequitable subgroups of digital mental health relational agent users, the novel study was not without major limitations, including, first and foremost, the exploratory, single-arm design that precludes a true evaluation of differences in efficacy across different subpopulations. The bivariate analyses did not account for differences in demographic or clinical characteristics across residential subgroups because of the exploratory nature of the analyses. In addition, rural populations had almost no representation (n=3) in the study, limiting the generalizability of the findings. Despite improvements that have narrowed the access gap in recent years, rural residents are less likely to own a smartphone than urban residents (80% vs 89%, respectively) and more likely to report access to high-speed internet as a major problem (24% vs 13%) [37]. Additional investigations targeting these important subgroups are urgently needed to determine if the findings of largely equitable outcomes across MUAs and MHPSAs extend to those living in rural areas. Ideally, future study designs should include a comparison group to establish whether these preliminary study findings persist in more rigorous randomized controlled trial environments.

In summary, this study’s findings suggest that, when accessed, vulnerable residential populations might benefit from access to digital mental health agent-guided cognitive behavioral therapy in ways similar to those living in more equitable regions. Programs like WB-LIFE could therefore represent an important public health opportunity.

## Appendix

Multimedia Appendix 1 Table S1. App use, therapeutic alliance, mental health outcomes, and satisfaction by residential subgroup in an 8-week, single-arm trial of a convenience sample of US adults aged 18 years and older.

## Abbreviations

DMHI: digital mental health intervention
GAD-7: Generalized Anxiety Disorder-7 item
MHPSA: mental health provider shortage area
MUA: medically underserved area
PHQ-8: Patient Health Questionnaire-8 item scale
WB-LIFE: Woebot LIFE

## Data Availability

The data sets generated during and analyzed during this study are not publicly available due to their proprietary nature but are available from the corresponding author on reasonable request.

## References

[1] Rehm J, Shield KD (2019). Global burden of disease and the impact of mental and addictive disorders. Curr Psychiatry Rep.

[2] (2017). Depression and other common mental disorders: global health estimates. World Health Organization.

[3] (2022). COVID-19 pandemic triggers 25% increase in prevalence of anxiety and depression worldwide. World Health Organization.

[4] Aboujaoude E, Gega L, Parish MB, Hilty DM (2020). Editorial: digital interventions in mental health: current status and future directions. Front Psychiatry.

[5] Clarke G, Yarborough BJ (2013). Evaluating the promise of health IT to enhance/expand the reach of mental health services. Gen Hosp Psychiatry.

[6] Mongelli F, Georgakopoulos P, Pato MT (2020). Challenges and opportunities to meet the mental health needs of underserved and disenfranchised populations in the United States. Focus (Am Psychiatr Publ).

[7] Snell-Rood C, Hauenstein E, Leukefeld C, Feltner F, Marcum A, Schoenberg N (2017). Mental health treatment seeking patterns and preferences of Appalachian women with depression. Am J Orthopsychiatry.

[8] Jameson JP, Blank MB (2007). The role of clinical psychology in rural mental health services: defining problems and developing solutions. Clin Psychol Sci Pract.

[9] Lattie EG, Stiles-Shields C, Graham AK (2022). An overview of and recommendations for more accessible digital mental health services. Nat Rev Psychol.

[10] Firth J, Torous J, Nicholas J, Carney R, Rosenbaum S, Sarris J (2017). Can smartphone mental health interventions reduce symptoms of anxiety? A meta-analysis of randomized controlled trials. J Affect Disord.

[11] Firth J, Torous J, Nicholas J, Carney R, Pratap A, Rosenbaum S, Sarris J (2017). The efficacy of smartphone-based mental health interventions for depressive symptoms: a meta-analysis of randomized controlled trials. World Psychiatry.

[12] Andrews G, Cuijpers P, Craske MG, McEvoy P, Titov N (2010). Computer therapy for the anxiety and depressive disorders is effective, acceptable and practical health care: a meta-analysis. PLoS One.

[13] Linardon J, Cuijpers P, Carlbring P, Messer M, Fuller-Tyszkiewicz M (2019). The efficacy of app-supported smartphone interventions for mental health problems: a meta-analysis of randomized controlled trials. World Psychiatry.

[14] Bower P, Kontopantelis E, Sutton A, Kendrick T, Richards DA, Gilbody S, Knowles S, Cuijpers P, Andersson G, Christensen H, Meyer B, Huibers M, Smit F, van Straten A, Warmerdam L, Barkham M, Bilich L, Lovell K, Liu ETH (2013). Influence of initial severity of depression on effectiveness of low intensity interventions: meta-analysis of individual patient data. BMJ.

[15] Wright JH, Mishkind M, Eells TD, Chan SR (2019). Computer-assisted cognitive-behavior therapy and mobile apps for depression and anxiety. Curr Psychiatry Rep.

[16] Designated health professional shortage areas statistics. Health Resources and Services Administration (HRSA).

[17] Vallury KD, Jones M, Oosterbroek C (2015). Computerized cognitive behavior therapy for anxiety and depression in rural areas: a systematic review. J Med Internet Res.

[18] Kay-Lambkin FJ, Baker AL, Kelly BJ, Lewin TJ (2012). It's worth a try: the treatment experiences of rural and urban participants in a randomized controlled trial of computerized psychological treatment for comorbid depression and alcohol/other drug use. J Dual Diagn.

[19] Fitzpatrick KK, Darcy A, Vierhile M (2017). Delivering cognitive behavior therapy to young adults with symptoms of depression and anxiety using a fully automated conversational agent (Woebot): a randomized controlled trial. JMIR Ment Health.

[20] Inkster B, Sarda S, Subramanian V (2018). An empathy-driven, conversational artificial intelligence agent (Wysa) for digital mental well-being: real-world data evaluation mixed-methods study. JMIR Mhealth Uhealth.

[21] Prochaska JJ, Vogel EA, Chieng A, Kendra M, Baiocchi M, Pajarito S, Robinson A (2021). A therapeutic relational agent for reducing problematic substance use (Woebot): development and usability study. J Med Internet Res.

[22] Munder T, Wilmers F, Leonhart R, Linster HW, Barth J (2010). Working Alliance Inventory-Short Revised (WAI-SR): psychometric properties in outpatients and inpatients. Clin Psychol Psychother.

[23] Larsen DL, Attkisson CC, Hargreaves WA, Nguyen TD (1979). Assessment of client/patient satisfaction: development of a general scale. Eval Program Plann.

[24] Kroenke K, Strine TW, Spitzer RL, Williams JBW, Berry JT, Mokdad AH (2009). The PHQ-8 as a measure of current depression in the general population. J Affect Disord.

[25] Spitzer RL, Kroenke K, Williams JBW, Löwe B (2006). A brief measure for assessing generalized anxiety disorder: the GAD-7. Arch Intern Med.

[26] Cohen S, Kamarck T, Mermelstein R (1994). Perceived stress scale. Measuring Stress: A Guide for Health and Social Scientists.

[27] Smith BW, Dalen J, Wiggins K, Tooley E, Christopher P, Bernard J (2008). The brief resilience scale: assessing the ability to bounce back. Int J Behav Med.

[28] Dolan ED, Mohr D, Lempa M, Joos S, Fihn SD, Nelson KM, Helfrich CD (2015). Using a single item to measure burnout in primary care staff: a psychometric evaluation. J Gen Intern Med.

[29] McMurray JE, Linzer M, Konrad TR, Douglas J, Shugerman R, Nelson K (2000). The work lives of women physicians results from the physician work life study. The SGIM career satisfaction study group. J Gen Intern Med.

[30] Find shortage areas by address. Health Resources and Services Administration (HRSA).

[31] (2020). Rural-urban continuum codes. USDA Economic Research Service.

[32] (2020). Rural-urban continuum codes documentation. USDA Economic Research Service.

[33] McGranahan DA, Hession JC, Mines FK, Jordan MF (1986). Social and economic characteristics of the population in metro and nonmetro counties, 1970-80. AgEcon SEARCH: Research in Agricultural & Applied Economics.

[34] Chiauzzi E, Williams A, Mariano TY, Pajarito S, Robinson A, Kirvin-Quamme A, Forman-Hoffman V Demographic and clinical characteristics associated with anxiety and depressive symptom outcomes in users of a digital mental health intervention incorporating a relational agent. Research Square..

[35] Wang PS, Lane M, Olfson M, Pincus HA, Wells KB, Kessler RC (2005). Twelve-month use of mental health services in the United States: results from the National Comorbidity Survey Replication. Arch Gen Psychiatry.

[36] Blanco C, Okuda M, Markowitz JC, Liu SM, Grant BF, Hasin DS (2010). The epidemiology of chronic major depressive disorder and dysthymic disorder: results from the National Epidemiologic Survey on Alcohol and Related Conditions. J Clin Psychiatry.

[37] Vogels EA (2021). Some digital divides persist between rural, urban and suburban America. Pew Research Center.

