# Supplementary material for: Engagement, Satisfaction, and Mental Health Outcomes Across Different Residential Subgroup Users of a Digital Mental Health Relational Agent: Exploratory Single-Arm Study
Source: JMIR Form Res. 2023 Sep 27;7:e46473. doi: 10.2196/46473 (PMC10568381; doi:10.2196/46473)
Supplement: Multimedia Appendix 1 [file formative_v7i1e46473_app1.docx]

**Table S1**. App use, therapeutic alliance, mental health outcomes, and satisfaction by residential subgroup in an 8-week, single-arm trial of a convenience sample of US adults aged 18 years and above.

|  | **Residential Characteristic** | | | | | |
| --- | --- | --- | --- | --- | --- | --- |
| **Outcome**  **M(sd)** | **MUA No**  **N=156** | **MUA Yes**  **N=99** | **Test statistic, effect size** | **MHPSA No**  **N=114** | **MHPSA Yes**  **N=141** | **Test statistic, effect size** |
| Utilization | | | | | | |
| Opened app on at least half of the weeks (4 of 8) (n, %) | 118/156 (76%) | 71/99 (72%) | Fisher’s test OR = 0.82 (0.45, 1.51), p=.56 | 81/114 (71%) | 108/141 (77%) | Fisher’s test OR = 1.33 (0.73, 2.43), p=.32 |
| Therapeutic Alliance | | | | | | |
| Goal at Day 3 | **3.40 (1.18)** | **3.73 (1.12)** | **t=-2.21, df=197.51, p=.03, Cohen’s d=-.29 (-.55, -.03)** | 3.52 (1.19) | 3.53 (1.15) | t=-.03, df=229.31, p=.97, Cohen’s d=.00 (-.26, .25) |
|  | **Mean difference = -0.33 (-0.64, -0.04)** | |  | Mean difference = -0.01 (-0.30, 0.29) | |  |
| Goal at Week 8 | **3.59 (1.17)** | **3.94 (1.13)** | **t=-2.28, df=186.19, p=.02, Cohen’s d=-.31 (-.57, -.04)** | 3.63 (1.24) | 3.80 (1.10) | t=-1.09, df=215.82, p=.28, Cohen’s d=-.14 (-.40, .11) |
|  | **Mean difference = -0.35 (-0.66, -0.05)** | |  | Mean difference = -0.17 (-0.47, 0.14) | |  |
| Task at Day 3 | 3.25 (1.02) | 3.42 (1.10) | t=-1.16, df=178.70, p=.25, Cohen’s d=-.16 (-.42, .10) | 3.32 (1.09) | 3.32 (1.01) | t=-0.01, df=230.15, p=.99, Cohen’s d=.00 (-.25, .25) |
|  | Mean difference = -0.17 (-0.44, 0.11) | |  | Mean difference = 0.00 (-0.27, 0.27) | |  |
| Task at Week 8 | 3.49 (0.98) | 3.61 (1.10) | t=-0.82, df=168.03, p=.41, Cohen’s d=-.11 (-.38, .15) | 3.51 (0.99) | 3.55 (1.05) | t=-0.29, df=228.51, p=.77, Cohen’s d=-.04 (-.30, .22) |
|  | Mean difference = -0.12 (-0.40, 0.16) | |  | Mean difference = -0.04 (-0.30, 0.23) | |  |
| Bond at Day 3 | 3.77 (1.08) | 4.04 (0.99) | **t=-1.94, df=203.47, p=0.05, Cohen’s d=-.25 (-.51, .01)** | 3.94 (1.06) | 3.82 (1.04) | t=0.88, df=233.04, p=.38, Cohen’s d=.11 (-.14, .37) |
|  | Mean difference = -0.27 (-0.53, 0.004) | |  | Mean difference = 0.12 (-0.15, 0.39) | |  |
| Bond at Week 8 | 4.01 (1.08) | 4.12 (1.12) | t=-0.76, df=175.54, p=.45, Cohen’s d=-.10 (-.37, .16) | 4.16 (1.03) | 3.96 (1.15) | t=1.36, df=228.91, p=.18, Cohen’s d=.18 (-.08, .44) |
|  | Mean difference = -0.11 (-0.41, 0.18) | |  | Mean difference = 0.20 (-0.09, 0.48) | |  |
| Total at Day 3 | **3.44 (1.01)** | **3.73 (0.99)** | **t=-2.15, df=192.98, p=.03, Cohen’s d=-.28 (-.54, -.02)** | 3.56 (1.04) | 3.54 (1.00) | t=0.11, df=233.34, p=.91, Cohen’s d=.01 (-.24, .27) |
|  | **Mean difference = -0.29 (-0.55, -0.02)** | |  | Mean difference = 0.02 (-0.24, 0.27) | |  |
| Total at Week 8 | 3.70 (0.95) | 3.86 (1.08) | t=-1.18, df=166.54, p=.24, Cohen’s d=-.16 (-.43, .10) | 3.77 (0.96) | 3.75 (1.04) | t=0.12, df=229.11, p=.90, Cohen’s d=.02 (-.24, .27) |
|  | Mean difference = -0.16 (-0.44, 0.11) | |  | Mean difference = 0.02 (-0.24, 0.28) | |  |
| Mental Health Outcome | | | | | | |
| Depressive Symptoms (PHQ-8) at baseline | 8.87 (5.97) | 9.53 (6.98) | t=-0.78, df=185.08, p=.44, Cohen’s d=-.10 (-.36, .15) | 9.25 (5.91) | 9.02 (6.75) | t=0.28, df=251.34, p=.78, Cohen’s d=.04 (-.21, .28) |
|  | Mean difference = -0.66 (-2.33, 1.01) | |  | Mean difference = 0.23 (-1.34, 1.79) | |  |
| Depressive Symptoms at Week 8 | 5.68 (4.75) | 5.43 (5.00) | t=0.38, df=176.37, p=.71, Cohen’s d=.05 (-.21, .32) | 5.39 (4.88) | 5.76 (4.81) | t=-0.57, df=226.22, p=.57, Cohen’s d=-.08 (-.33, .18) |
|  | Mean difference = 0.25 (-1.06, 1.56) | |  | Mean difference = -0.37 (-1.62, 0.98) | |  |
| Change in depressive symptoms at Week 8 | -3.19 (5.45) | -4.17 (6.24) | t=1.22, df=164.79, p=.22, Cohen’s d=.17 (-.09, .44) | -3.83 (5.89) | -3.32 (5.67) | t=-0.66, df=224.79, p=.51, Cohen’s d=-.09 (-.34, .17) |
|  | Mean difference = 0.98 (-0.60, 2.57) | |  | Mean difference = -0.51 (-2.00, 0.99) | |  |
| Depressive Symptoms (PHQ-8) at baseline^a^ | **14.41 (3.97)**  **n=68** | **16.50 (4.13)**  **n=42** | **t=-2.62, df=84.40, p=.01, Cohen’s d=-.52 (-.91, -.13)** | 15.06 (3.99)  n=47 | 15.32 (4.28)  n=63 | t=-0.32, df=102.76, p=.75, Cohen’s d=-.06 (-.44, .32) |
|  | **Mean difference = -2.09 (-3.68, -0.50)** | |  | Mean difference = -0.26 (-1.83, 1.32) | |  |
| Depressive Symptoms at Week 8^a^ | 7.97 (4.63) | 8.21 (5.26) | t=-0.23, df=70.22, p=.82, Cohen’s d=-.05 (-.45, .35) | 7.55 (5.28) | 8.45 (4.50) | t=-0.91, df=84.12, p=.37, Cohen’s d=-.19 (-.58, .21) |
|  | Mean difference = -0.24 (-2.30, 1.81) | |  | Mean difference = -0.90 (-2.88, 1.07) | |  |
| 8-week change in depressive symptoms^a^ | -6.41 (5.29) | -8.39 (6.39) | t=1.62, df=66.78, p=.11, Cohen’s d=.35 (-.06, .75) | -7.66 (6.24) | -6.76 (5.42) | t=-0.76, df=85.22, p=.45, Cohen’s d=-.16 (-.55, .24) |
|  | Mean difference = 1.98 (-0.47, 4.44) | |  | Mean difference = -0.90 (-3.25, 1.45) | |  |
| Anxiety Symptoms (GAD-7) at baseline | 8.76 (5.43) | 8.83 (6.39) | t=-0.09, df=183.97, p=.93, Cohen’s d=-.01 (-.26, .24) | 9.28 (5.55) | 8.38 (6.00) | t=1.24, df=248.45, p=.22, Cohen’s d=.15 (-.09, .40) |
|  | Mean difference = -0.07 (-1.60, 1.46) | |  | Mean difference = 0.90 (-0.53, 2.33) | |  |
| Anxiety Symptoms at Week 8 | 5.14 (4.62) | 4.83 (4.48) | t=0.51, df=188.39, p=.61, Cohen’s d=.07 (-.20, .33) | 5.13 (4.82) | 4.94 (4.34) | t=0.32, df=218.96, p=.75, Cohen’s d=.04 (-.22, .30) |
|  | Mean difference = 0.31 (-0.89, 1.52) | |  | Mean difference = 0.19 (-1.00, 1.38) | |  |
| Change in anxiety symptoms at Week 8 | -3.68 (5.15) | -4.12 (5.92) | t=0.59, df=164.51, p=.56, Cohen’s d=.08 (-.18, .35) | -4.28 (5.45) | -3.47 (5.44) | t=-1.13, df=227.09, p=.26, Cohen’s d=-.15 (-.41, .11) |
|  | Mean difference = 0.44 (-1.06, 1.95) | |  | Mean difference = -0.81 (-2.22, 0.60) | |  |
| Anxiety Symptoms (GAD-7) at baseline^b^ | 14.14 (3.49)  n=63 | 15.07 (3.73)  n=43 | t=-1.29, df=86.34, p=.20, Cohen’s d=-.26 (-.65, .13) | 14.46 (3.66)  n=50 | 14.57 (3.57)  n=56 | t=-0.16, df=102.03, p=.87, Cohen’s d=-.03 (-.41, .35) |
|  | Mean difference = -0.93 (-2.35, 0.50) | |  | Mean difference = -0.11 (-1.51, 1.29) | |  |
| Anxiety Symptoms at Week 8^b^ | 7.41 (5.10) | 7.21 (5.04) | t=0.19, df=79.81, p=.85, Cohen’s d=.04 (-.37, .45) | 7.65 (5.39) | 7.00 (4.71) | t=0.64, df=93.82, p=.53, Cohen’s d=.13 (-.27, .53) |
|  | Mean difference = 0.20 (-1.90, 2.29) | |  | Mean difference = 0.65 (-1.39, 2.69) | |  |
| Change in anxiety symptoms at Week 8^b^ | -6.76 (5.62) | -8.18 (6.25) | t=1.14, df=72.96, p=.26, Cohen’s d=.24 (-.17, .65) | -6.80 (6.44) | -7.85 (5.27) | t=0.89, df=92.10, p=.38, Cohen’s d=.18 (-.22, .58) |
|  | Mean difference = 1.42 (-1.07, 3.91) | |  | Mean difference = 1.05 (-1.31, 3.43) | |  |
| Stress (PSS) at baseline | 21.29 (5.04) | 21.38 (6.41) | t=-0.13, df=173.20, p=.90, Cohen’s d=-.02 (-.27, .23) | 22.13 (4.88) | 20.67 (6.06) | **t=2.13, df=253.00, p=.03, Cohen’s d=.26 (.01, .51)** |
|  | Mean difference = -0.09 (-1.60, 1.41) | |  | Mean difference = 1.46 (0.11, 2.81) | |  |
| Stress (PSS) at Week 8 | 18.84 (5.87) | 18.58 (5.97) | t=0.33, df=181.25, p=.74, Cohen’s d=.04 (-.22, .31) | 19.04 (5.90) | 18.48 (5.90) | t=0.71, df=227.15, p=.48, Cohen’s d=.09 (-.16, .35) |
|  | Mean difference = 0.26 (-1.32, 1.84) | |  | Mean difference = 0.56 (-0.97, 2.08) | |  |
| Change in stress at Week 8 | -2.46 (6.94) | -2.72 (6.14) | t=0.30, df=201.30, p=.77, Cohen’s d=.04 (-.23, .30) | -3.20 (7.14) | -1.98 (6.14) | t=-1.39, df=214.39, p=.17, Cohen’s d=-.18 (-.44, .07) |
|  | Mean difference = 0.26 (-1.46, 1.98) | |  | Mean difference = -1.22 (-2.95, 0.51) | |  |
| Resilience (BRS) at baseline | 2.93 (0.86) | 3.02 (0.91) | t=-0.72, df=200.72, p=.47, Cohen’s d=-.09 (-.35, .16) | 2.92 (0.85) | 3.00 (0.90) | t=-.76, df=246.22, p=.45, Cohen’s d=-.09 (-.34, .15) |
|  | Mean difference = -0.09 (-0.31, 0.14) | |  | Mean difference = 0.08 (-0.30, 0.13) | |  |
| Resilience (BRS) at Week 8 | 3.30 (0.87) | 3.28 (0.91) | t=0.17, df=179.53, p=.86, Cohen’s d=.02 (-.24, .29) | 3.27 (0.87) | 3.31 (0.89) | t=-0.33, df=230.08, p=.74, Cohen’s d=-.04 (-.30, .21) |
|  | 0.02 (-0.21, 0.26) | |  | Mean difference = -0.04 (-0.27, 0.19) | |  |
| Change in resilience at Week 8 | 0.36 (0.74) | 0.25 (0.73) | t=1.13, df=188.55, p=.26, Cohen’s d=.15 (-.11, .41) | 0.36 (0.72) | 0.29 (0.75) | t=0.73, df=231.14, p=.47, Cohen’s d=.10 (-.16, .35) |
|  | Mean difference = 0.11 (-0.08, 0.31) | |  | Mean difference = 0.07 (-0.12, 0.26) | |  |
| Burnout at baseline (n, %) | 95/155 (61%) | 56/99 (57%) | Fisher’s test OR = 0.82 (0.48, 1.42), p=.52 | 70/113 (62%) | 81/141 (57%) | Fisher’s test OR = 0.83 (0.48, 1.42), p=.52 |
| Burnout at Week 8 (n, %) | 55/144 (38%) | 32/88 (36%) | Fisher’s test OR = 0.93 (0.51, 1.66), p=.89 | 38/108 (35%) | 49/124 (40%) | Fisher’s test OR = 1.20 (0.68, 2.13), p=.59 |
| Improvement of burnout at Week 8^c^ (n, %) | 46/88 (52%) | 27/50 (54%) | Fisher’s test OR = 0.93 (0.44, 1.98), p=.86 | 37/67 (55%) | 36/71 (51%) | Fisher’s test OR = 1.20 (0.58, 2.47), p=.61 |
| Satisfaction | | | | | | |
| CSQ at Week 8 | 25.84 (5.40) | 26.66 (5.24) | t=-1.14, df=188.18, p=.25, Cohen’s d=-.15 (-.42, .11) | 25.84 (5.22) | 26.42 (5.46) | t=-0.82, df=229.37, p=.41, Cohen’s d=-.11 (-.37, .15) |
|  | Mean difference = -0.82 (-2.23, 0.59) | |  | Mean difference = -0.58 (-1.95, 0.80) | |  |

BRS-Brief Resilience Scale; GAD-7=Generalized Anxiety Disorder-7 item scale; HPSA=mental health provider shortage area; MHPSA=mental health provider shortage areas; MUA=medically underserved area; PHQ-8=Patient Health Questionnaire-8 item scale; PSS=Perceived Stress Scale; WAI=Working Alliance Inventory

^a^ among users with PHQ-8≥10 at baseline

^b^ among users with GAD-7≥10 at baseline

^c^ among users with burnout at baseline
